# Pediatric anaphylaxis: age-related symptom trends and the limited role of allergen molecules: a retrospective analysis

**DOI:** 10.1186/s13223-025-01000-2

**Published:** 2025-11-27

**Authors:** Izabela Kucharek, Krzysztof Przystał-Dyszyński, Aleksandra Godyńska, Maria Gregorczyk, Natasza Krajewska, Adam J. Sybilski

**Affiliations:** Clinical Department of Pediatrics and Allergology, National Medical Institute of the Ministry of the Interior and Administration, Warsaw, Poland

**Keywords:** Anaphylaxis, Phenotype, Age Factors, Child, Allergen components, Component-resolved diagnostics

## Abstract

**Background:**

Emerging evidence suggests that specific allergen molecules may influence the clinical phenotype of anaphylaxis in children, but robust data are scarce. This study aimed to rigorously test the molecule-phenotype association in a large pediatric cohort and to determine the relative influence of the sensitizing molecule versus patient age on symptom presentation.

**Methods:**

A retrospective analysis was conducted on 184 pediatric patients (0–18 years) hospitalized for anaphylaxis. Molecular allergen-specific immunoglobulin E (IgE) profiles were determined using the ALEX^2^ test. Symptom frequencies across different organ systems were analyzed in relation to allergen molecules and age groups using Cochran’s Q and Pearson’s χ2 tests.

**Results:**

The most frequent molecular triggers were Ara h 2 (18.79%), Gal d 1 (9.09%), and Ana o 3 (9.09%). While significant differences in symptom distribution were observed within individual allergen molecules (*p* < 0.05), no molecule-specific symptom pattern was identified. In contrast, age significantly influenced respiratory symptom prevalence, with a higher frequency in older children compared to infants (*p* = 0.003). A similar trend was observed for gastrointestinal symptoms (*p* = 0.051).

**Conclusions:**

In pediatric anaphylaxis, patient age is a more significant determinant of clinical presentation, particularly for respiratory symptoms, than the specific sensitizing allergen molecule. This suggests that clinical risk stratification and management strategies in children should prioritize age-related factors over specific molecular sensitization profiles.

**Supplementary Information:**

The online version contains supplementary material available at 10.1186/s13223-025-01000-2.

## Introduction

Anaphylaxis is a severe, life-threatening hypersensitivity reaction of sudden onset that involves various organ systems and requires immediate medical intervention. It represents a significant clinical challenge, especially in the pediatric population. Hospital admissions for anaphylaxis have increased in recent decades, mainly due to food, drug, and Hymenoptera venom allergy [1]. Allergy diagnosis has evolved significantly over time. Historically, diagnosis was based on tests using allergen extracts. However, molecular diagnosis now allows for the assessment of sensitization to specific allergen components. This approach facilitates the identification of primary allergenic proteins, the distinction of cross-reactivity among allergens, the enhancement of accurate allergen detection, the reduction of the risk of severe allergic reactions, and the guidance of allergen immunotherapy. [2] Few studies using molecular diagnostics have focused primarily on assessing the severity of reactions caused by exposure to specific molecules [3]. Preliminary findings suggest that certain allergen molecules may be associated with distinct symptom profiles, particularly involving respiratory or cardiovascular systems [4] Precision medicine in allergology aims to identify disease phenotypes and endotypes, which allows tailoring diagnosis and treatment to the individual molecular mechanisms responsible for the development of reactions (5). The present retrospective study aimed to assess the clinical and molecular factors associated with the occurrence of anaphylaxis in children hospitalized at our center by analyzing data on 184 pediatric patients who were tested for specific IgE antibodies to 178 allergen molecules. We focused on identifying the molecules responsible for triggering anaphylactic reactions and the possible specific clinical symptom profiles associated with exposure to a given molecule. We also considered demographic variables and clinical factors of the study group.

## Methods

### Study design

The study was retrospective in nature and was conducted at the Department of Pediatrics and Allergology of the National Medical Institute of the Ministry of the Interior and Administration, Warsaw, Poland (PIM MSWiA). The analysis included medical data from 184 pediatric patients aged between 0 and 18 years who were hospitalized for anaphylactic reactions between 2020 and 2024.

### Inclusion and exclusion criteria

Patients aged 0 to 18 years, hospitalized in the Department of Pediatrics and Allergology of PIM MSWiA for documented anaphylactic reactions, with available allergen molecular diagnostic results and complete medical records, including detailed demographic data, history of atopic diseases and information on the course of anaphylaxis, were included in the study. Patients not meeting the above criteria were excluded from the study. The study included all pediatric patients hospitalized for anaphylaxis at our center between 2020 and 2024. The sample size was determined by the total number of eligible cases available in the hospital database during this period, without preselection or additional inclusion criteria.

### Study procedure

Data were collected from patients' medical records and anonymized by the research team. Information included:

- Demographic data,
- Potential triggers of anaphylaxis based on history,
- Clinical history, including atopic diseases (atopic dermatitis, allergic asthma), family history of allergy, and number of previous episodes of anaphylaxis,
- Symptoms of anaphylaxis, including reactions from the cardiovascular system, respiratory system, skin, gastrointestinal system and nervous system. Furthermore, symptoms that did not align with the above-mentioned categories were analyzed.
- Molecular test results for 178 allergen molecules.

### Molecular diagnostics

Molecular diagnostics were performed using the ALEX^2^ (Allergy Explorer) test (MacroArray Diagnostics, Vienna, Austria), according to the procedure described by the manufacturer. The ALEX^2^ test allows simultaneous determination of specific IgE against 295 allergens, including 178 molecular components and 117 allergen extracts.

### Definitions and grading system

Anaphylaxis was defined in accordance with the European Academy of Allergy and Clinical Immunology (EAACI) guidelines [1]. The severity of anaphylaxis was assessed based on the updated grading system for systemic allergic reactions proposed by the World Allergy Organization Anaphylaxis Committee and Allergen Immunotherapy Committee. Brief descriptors are provided in Supplementary Table S1, with full criteria in the original publication [6].

### Data analysis

In cases of polysensitization, the decision to attribute an anaphylactic reaction to a particular molecule was based on clinical history, the profile of molecular diagnostic findings and the known anaphylactogenicity of individual molecules. The most likely allergen was determined based on the highest level of specific IgE combined with reports of the molecules' potential to cause immediate reactions. Age was analyzed as a categorical variable, divided into the following groups: 0–11 months, 1–5 years, and 6–11 years, following commonly used pediatric age classifications to ensure consistency with previous research.

Analysis of differences in the frequency of anaphylactic signs and symptoms from different systems was performed for each allergen molecule that met the requirements (number of observations for the molecule was greater than or equal to 5 and the number of observations that differed in at least 1 system was greater than 4). An initial analysis was performed on all systems using Cochran's Q test. For molecules that showed a significant difference in frequencies, individual systems were compared using the exact McNemar's test.

Analyses of differences in the frequency of anaphylaxis signs and symptoms as a function of allergen molecule and age were performed for each system that met the requirements (expected frequencies greater than or equal to 5 and number of observations greater than 13). Initial analysis was performed on the entire table using Pearson's χ2 test. For systems that showed a significant difference in frequencies, individual groups were compared pairwise, with the significance level adjusted for the number of comparisons.

For all the above analyses, calculations were performed using the Python 3.12.9 programming language with the following modules: statsmodels 0.14.4, scipy 1.14.1, pandas 2.2.3. Sensitivity analyses were not conducted, as the study design and data structure did not warrant additional validation through reanalysis.

## Results

Of the 184 patients, the trigger for the anaphylactic reaction at the molecular level was determined for 165. Baseline demographics and clinical characteristics are shown in Table 1. A consolidated summary of eliciting factors together with their most frequent molecular components is provided in Table 2.

**Table 1:** Demographic and Clinical Characteristics of the Study Population

Variable	Value
Number of patients	184
Age (months), median (range)	29 (6–208)
Sex, n (%)	
Male	118 (64.1)
Female	66 (35.9)
Family history of allergy, n (%)	
Positive	85 (46.2)
Negative	92 (50.0)
Missing data	7 (3.8)
Atopic dermatitis, n (%)	
None	103 (56.0)
Mild	62 (33.7)
Moderate	18 (9.8)
Severe	1 (0.5)
Asthma, n (%)	
None	143 (77.7)
Mild	35 (19.0)
Moderate	6 (3.3)
Severe	0 (0.0)
Number of previous anaphylaxis episodes, n (%)	
0 episodes	117 (63.6)
1 episode	40 (21.7)
≥ 2 episodes	27 (14.7)
Anaphylaxis severity (WAO scale), n (%)	
Grade 1	8 (4.4)
Grade 2	55 (29.9)
Grade 3	111 (60.3)
Grade 4	9 (4.9)
Grade 5	1 (0.5)
Anaphylaxis symptoms, n (%)	
Cutaneous	182 (98.9)
Respiratory	107 (58.2)
Gastrointestinal	91 (49.5)
Cardiovascular	11 (6.0)
Neurological	43 (23.4)

n, Number, %, Percentage, WAO, World Allergy Organization

**Table 2:** Eliciting factors with top molecular components

Eliciting factor	n (all)	% (all)	Top molecule 1	Top molecule 2	Top molecule 3
Peanut	44	26.7	Ara h 2 (n = 31)	Ara h 6 (n = 7)	Ara h 1 (n = 5)
Hen’s egg	25	15.2	Gal d 1 (n = 16)	Gal d 2 (n = 5)	Gal d 4 (n = 2)
Cow’s milk	17	10.3	Bos d 8 (n = 15)	Bos d 4 (n = 1)	Bos d 5 (n = 1)
Cashew	15	9.1	Ana o 3 (n = 15)		
Sesame	12	7.3	Ses i 1 (n = 12)		
Hazelnut	11	6.7	Cor a 14 (n = 8)	Cor a 11 (n = 2)	Cor a 9 (n = 1)
Walnut	10	6.1	Jug r 1 (n = 10)		
Wheat	6	3.6	Tri a 19 (n = 4)	Tri a 14 (n = 2)	
Fish	5	3.0	Gad m 1 (n = 4)	Cyp c 1 (n = 1)	
Kiwi	4	2.4	Act d 1 (n = 2)	Act d 2 (n = 1)	Act d 5 (n = 1)
Soy	4	2.4	Gly m 4 (n = 4)		
Shrimp	3	1.8	Pen m 4 (n = 2)	Pen m 1 (n = 1)	
Pea	2	1.2	Pis s 1 (n = 2)		
Pistachio	2	1.2	Pis v 1 (n = 2)		
Brazil nut	1	0.6	Ber e 1 (n = 1)		
Carrot	1	0.6	Dau c 1 (n = 1)		
Pork	1	0.6	Sus s 1 (n = 1)		
Pumpkin	1	0.6	Cuc p 2 (n = 1)		
Wasp venom	1	0.6	Ves v 5 (n = 1)		

Table shows all eliciting factors with counts and percentages in the full cohort, together with up to three most frequent molecular components per factor (WHO/IUIS nomenclature). Numbers reflect episodes in the analytic dataset

Total N = 165. Abbreviations: n, number; %, percentage; WHO, World Health Organization; IUIS, International Union of Immunological Societies.

### Analysis of differences in symptom frequency by allergen molecule

The analysis revealed statistically significant differences in the frequency of anaphylaxis symptoms across different organ systems within each allergen molecule (Cochran’s Q test, *p* < 0.05 for all analyzed molecules). However, no statistically significant differences were found in symptom distribution between different allergen molecules (χ^2^ test, *p* > 0.05).

**Fig. 1:**
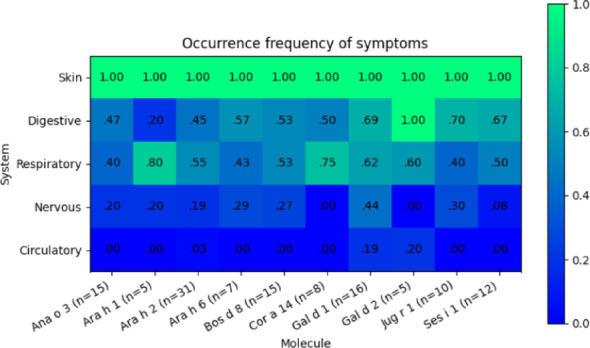
Occurrence frequency of symptoms by allergen molecule. The heatmap illustrates the prevalence of symptoms across different organ systems. Data are presented as the proportion of cases with a given symptom for each allergen molecule. The color gradient represents the proportion of affected individuals, with higher frequencies indicated by green and lower frequencies by blue. See Table 2 for the corresponding eliciting factors and predominant components

**Fig. 2:**
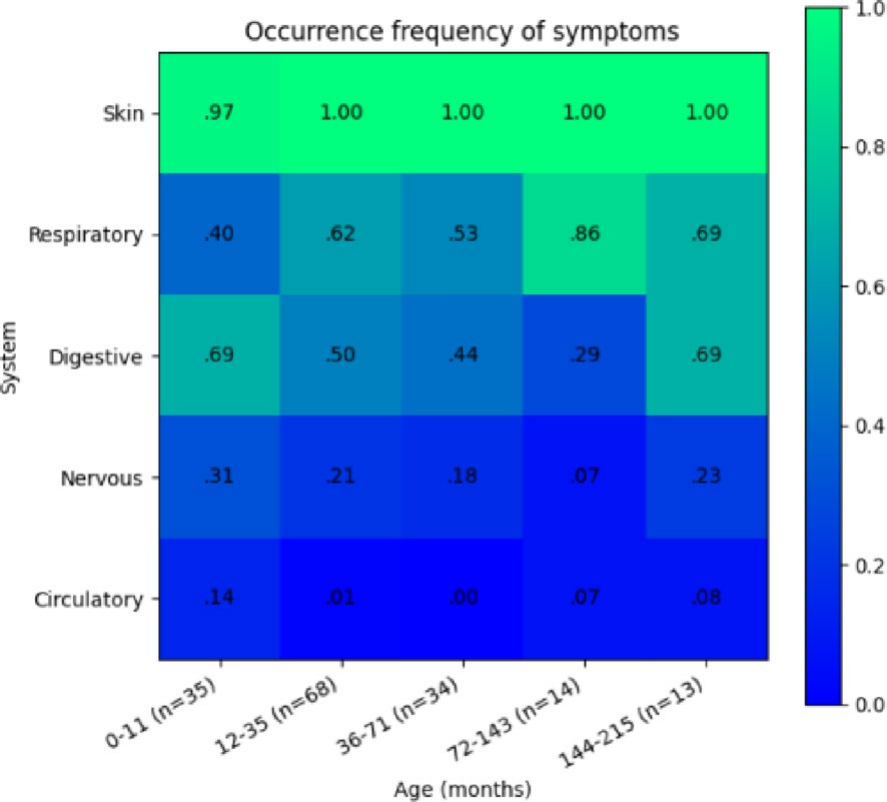
Occurrence frequency of symptoms by age. Heatmap illustrating the occurrence frequency of symptoms across different organ systems in children, categorized by age groups (in months). The color gradient represents the proportion of affected individuals, with higher frequencies indicated by green and lower frequencies by blue

The heatmap provides a visual representation of the symptom occurrence frequency, illustrating the general trend in the distribution of symptoms: Skin symptoms were present in all cases (100 percent), regardless of the allergen molecule. Gastrointestinal and respiratory symptoms occurred with similar frequency, appearing more frequently than neurological symptoms but less frequently than skin symptoms. Neurological symptoms were rare, observed in a range of 0 to 44 percent of cases, depending on the allergen molecule. Cardiovascular symptoms were the least common, with occurrence close to 0 percent in most cases. (Figs. 1 and 2).

Analysis of symptom frequency differences between allergen molecules (excluding skin symptoms due to their uniform presence) revealed that: For cardiovascular and neurological systems, the sample size was too small to perform a reliable statistical analysis. However, preliminary data suggest the possibility of differences in cardiovascular symptoms depending on the allergen molecule, indicating the need for a larger sample size. For other systems, no statistically significant differences were found in symptom frequency between allergen molecules (χ^2^ test for individual systems).

These findings indicate that although differences in symptom frequencies across different organ systems were observed within individual molecules, these differences did not translate into a clear phenotypic distinction between allergen molecules. The data indicate that while symptom frequencies vary within individual molecules, no clear evidence was found to suggest that specific allergen molecules systematically determine the phenotypic presentation of anaphylaxis. Consequently, no specific allergen molecule has been identified as a determining factor in these variations.

### Analysis of differences in symptom frequency by age

Analysis of symptom frequency differences across age groups (excluding skin symptoms due to their uniform presence) revealed that: For cardiovascular and neurological systems, the sample size was too small to perform a reliable statistical analysis. However, preliminary data suggest the possibility of differences in cardiovascular symptoms depending on age, indicating the need for a larger sample size. For the respiratory system, a statistically significant difference was found in symptom occurrence among different age groups (χ^2^ = 10.49, *p* = 0.033). Post hoc analysis indicated a significant difference specifically between the 0–11 months and 6–11 years (72–143 months) groups (χ^2^ = 8.39, *p* = 0.003), with an adjusted significance threshold of 0.005 due to multiple comparisons (Bonferroni correction, α = 0.05/10). For the gastrointestinal system, *p* = 0.051, slightly above the significance threshold. This suggests the potential presence of differences that may not be detectable due to sample size limitations.

## Discussion

To date, studies on the influence of molecular allergens on the clinical presentation of allergic reactions have mainly focused on chronic diseases such as allergic rhinitis, asthma or atopic dermatitis [7], while analyses on anaphylaxis are scarce. Although these studies are concerned with a different manifestation of allergy than anaphylaxis, their results suggest the possibility of specific clinical patterns dependent on particular allergen molecules. Notably, previous research has investigated the anaphylactogenic potential of specific allergen molecules, identifying certain components as stronger predictors of severe allergic reactions [8–10] .The findings of our study suggest that while anaphylaxis symptoms exhibit significant differences across organ systems within individual allergen molecules, no clear evidence was found to indicate that specific allergen molecules systematically determine the anaphylaxis phenotype. Although cardiovascular and neurological symptoms were too infrequent to allow for reliable statistical analysis, preliminary data suggest that cardiovascular symptoms may vary depending on both the allergen molecule and patient age, highlighting the need for further investigation. To the best of our knowledge, only one study to date has specifically examined the relationship between allergen molecules and symptom patterns in anaphylaxis, suggesting that some components, such as Ana o 3, Tri a 19, and Cor a 9, may be more frequently associated with either respiratory or cardiovascular involvement [4]. However, these studies focused on different molecular allergens than those analyzed in our research, which may explain differences in findings. While their results highlight potential molecular influences on anaphylaxis presentation, our study did not identify a systematic association between individual allergen molecules and specific symptom profiles. This may suggest that the role of molecular allergens in shaping anaphylaxis phenotypes is complex and potentially modulated by additional factors, including co-sensitization, individual immune responses, and underlying atopic conditions. In our cohort, the most severe presentations were uncommon (WAO grades 4- 5: 5.4%; Table 1), consistent with population analyses reporting that fatal food anaphylaxis is rare in young children and that mortality peaks in adolescence and young adulthood [11, 12]. Given this severity distribution, age emerged as a key modifier of presentation. Age-related differences in respiratory symptoms were statistically significant, particularly between infants and older children. While gastrointestinal symptoms showed a potential trend toward age-related variation, the results did not reach statistical significance. These findings align with previous studies that have also reported age-related differences in anaphylaxis symptomatology. Notably, our findings regarding respiratory symptoms are consistent with prior research, which also identified a higher frequency of wheezing in school-aged children compared to younger age groups. Similarly, the trend observed in gastrointestinal symptoms corresponds with previous findings indicating that vomiting is more prevalent in infants than in older children [13–15]. It is important to note that this age-dependent phenotype has clinical implications: in emergency department-based cohorts, anaphylaxis in the youngest children is more often under-recognized and basic hemodynamic assessment (e.g., blood pressure) is obtained less consistently, which can delay definitive therapy despite multisystem involvement [14]; moreover, underuse of epinephrine in acute care has been documented overall in pediatric series [13]. However, one notable discrepancy between our results and previous studies concerns cutaneous symptoms. Earlier research demonstrated age-related differences in the prevalence of urticaria, with the highest frequency among infants and a decreasing trend in older children [13, 14]. In contrast, in our cohort cutaneous symptoms were nearly universal, which likely precluded detection of age-related variation and may reflect differences in patient selection or clinical setting. Consistency across independent emergency department cohorts further reinforces that the absence of respiratory signs should not lower clinical suspicion in the youngest patients and supports explicitly age-attuned recognition and timely epinephrine when standard diagnostic criteria are met [15]. Discharge planning should prioritize caregiver education tailored to infant presentations (e.g., sudden repetitive vomiting, generalized urticaria). These steps are intended to improve recognition and time-to-treatment across age groups while maintaining standard diagnostic thresholds.

It should be noted, however, that this study has some limitations. The retrospective, single-center design and the absence of external validation may limit the generalizability of the results. Limitations in sample size, particularly for cardiovascular and neurological symptoms, may have impacted the ability to detect potential differences. Additionally, in polysensitized patients, it was necessary to select a single molecule responsible for the anaphylactic reaction. This selection was based on available scientific knowledge of the molecules, clinical data and specific IgE concentrations. Nevertheless, in a small number of cases, the final selection may have been subject to the risk of misinterpretation.

## Conclusions

While significant differences in symptom frequencies were observed within individual allergen molecules, no clear molecule-specific symptom patterns were identified. This suggests that the molecular structure of allergens alone may not be the primary determinant of anaphylaxis presentation. Notably, age was found to influence the occurrence of respiratory symptoms, with a statistically significant difference observed between infants and older children. Additionally, our findings suggest a potential age-related trend in gastrointestinal symptoms of anaphylaxis. Taken together, these findings support a multifactorial view of anaphylaxis that integrates molecular diagnostics with clinical and demographic context. In practical terms, the age-dependent phenotype argues for heightened clinical vigilance in infants and toddlers-maintaining suspicion when systemic diagnostic criteria are otherwise met even in the absence of respiratory signs, ensuring consistent hemodynamic monitoring, timely intramuscular epinephrine, and tailored caregiver education at discharge. Future research on larger, multicenter cohorts is needed to confirm these associations and refine the precision of age and allergen-specific risk assessment in pediatric anaphylaxis.

## Supplementary Information

Below is the link to the electronic supplementary material.

Supplementary Material 1

## Data Availability

The dataset supporting the conclusions of this article is available from the corresponding author upon reasonable request.
