# Supplementary material for: Pediatric anaphylaxis: age-related symptom trends and the limited role of allergen molecules: a retrospective analysis
Source: Allergy Asthma Clin Immunol. 2025 Nov 27;21:54. doi: 10.1186/s13223-025-01000-2 (PMC12750875; doi:10.1186/s13223-025-01000-2)
Supplement: Supplementary file 1 — Supplementary Material 1 [file 13223_2025_1000_MOESM1_ESM.docx]

Supplementary Table S1. WAO-Systemic Allergic Reaction (WAO-SAR) severity grades - brief descriptors

| Grade | Brief descriptor |
| --- | --- |
| 1 | Mild manifestations, typically limited to a single organ system. |
| 2 | Moderate systemic involvement without objective hypotension or hypoxia. |
| 3 | Severe respiratory compromise and/or gastrointestinal involvement meeting WAO anaphylaxis criteria (no shock or arrest). |
| 4 | Life-threatening compromise (e.g., significant hypotension or severe respiratory involvement) without cardiorespiratory arrest. |
| 5 | Cardiorespiratory arrest or fatal outcome. |

Notes: Brief descriptors adapted from the WAO-SAR grading (2024 Joint Statement) [6]. For full definitions and criteria, see ref. [6].
